# Comparison of the Effect of BPA and Related Bisphenols on Membrane Integrity, Mitochondrial Activity, and Steroidogenesis of H295R Cells In Vitro

**DOI:** 10.3390/life14010003

**Published:** 2023-12-19

**Authors:** Nikola Štefunková, Hana Greifová, Tomáš Jambor, Katarína Tokárová, Lucia Zuščíková, Denis Bažány, Peter Massányi, Marcela Capcarová, Norbert Lukáč

**Affiliations:** Institute of Applied Biology, Faculty of Biotechnology and Food Sciences, Slovak University of Agriculture in Nitra, Tr. A. Hlinku 2, 949 76 Nitra, Slovakiapeter.massanyi@uniag.sk (P.M.);

**Keywords:** BPA, bisphenol, viability, steroidogenesis, endocrine disruption, progesterone, testosterone, estradiol, cortisol

## Abstract

Bisphenol A (BPA) is an endocrine-disruptive chemical that is widely utilized in the production of polycarbonate plastic and epoxy resin, which are used to make a wide range of consumer products, food and drink containers, and medical equipment. When the potential risk of BPA emerged, it was substituted by allegedly less harmful substitutes such as bisphenols S, F, B, and AF. However, evidence suggests that all bisphenols can have endocrine-disruptive effects, while the extent of these effects is unknown. This study aimed to determine effect of BPA, BPAF, BPB, BPF, and BPS on viability and steroidogenesis in human adrenocortical carcinoma cell line in vitro. The cytotoxicity of bisphenols was shown to be considerable at higher doses. However, at low concentrations, it improved viability as well as steroid hormone secretion, indicating that bisphenols have a biphasic, hormetic effect in biological systems. The results are consistent with the hypothesis that bisphenols selectively inhibit some steroidogenic enzymes. These findings suggest that bisphenols have the potential to disrupt cellular steroidogenesis in humans, but substantially more detailed and systematic research is needed to gain a better understanding of the risks associated with bisphenols and their endocrine-disrupting effect on humans and wildlife.

## 1. Introduction

Xenoestrogens, also known as endocrine-disrupting chemicals (EDCs), are substances that can mimic or block endogenous hormones and interfere with endocrine processes [1]. Synthetic EDCs have been heavily incorporated into the environment for several decades, exposing humans and animals to their effects. Bisphenol A (BPA) is the most significant man-made substance among synthetic xenoestrogens, with yearly output reaching 3.8 million tons [2]. BPA is used in the manufacturing of polycarbonate plastics, epoxy resins, polysulfone, polyester, vinyl ester resins, and some flame retardants due to its ability to improve the durability and flexibility of plastics and synthetic products [3,4].

BPA can be produced as white flakes, pills, or crystals [5]. BPA is moderately soluble in water (120–300 ppm at 25 °C), which allows it to dissipate in the wastewater from factories that produce BPA-based products. Because of BPA’s high dissociation constant (pKa = 10.29), alkaline pH makes it more soluble [6]. BPA is primarily utilized in the production of polycarbonates (65%), followed by the production of epoxy resins (28%), other resins, and flame retardants (7%) [5,7]. Polycarbonates are utilized in multiple products of everyday use including water bottles, baby feeding bottles, toys, thermal paper, household appliances, and medical equipment due to their ability to withstand high temperatures (up to 145 °C) [7]. Due to their capacity to tolerate heat and chemicals, epoxy resins are frequently utilized as internal protective coatings for food and beverage containers, adhesives, paints, and electrical and electronic laminates [5,6]. Because BPA is used in all of these industrial applications, it has become ubiquitous in the environment. Air [8], soil [9], sediment [10], water [11], food [12], and biota (including humans [13], wildlife, and aquatic species [5]) all contain BPA. Humans are most commonly exposed to BPA via food, inhalation, and skin absorption [14]. The majority of BPA migration from plastics to food occurs when the cans of food are heated for sterilization [5]. Dust exposure is another way people can be exposed to BPA [15]. BPA’s effects on humans include cardiovascular problems [16], reproductive problems [17], problems with the development of the mammary gland [18], low sperm production [19], fetal growth restriction [17], anxiety, depression [20], and obesity [21,22], as well as hormone-related cancers such as breast cancer or prostate cancer [19]. BPA has been linked to developmental defects, apoptosis, and endocrine disruption, according to extensive research [3,21,23,24,25,26,27,28,29,30,31]. According to multiple studies conducted across the world, BPA has been detected in the urine, blood, and bodily fluids of 95% of individuals examined [3,4,23,24,25,26,27,28,32,33,34,35,36,37]. Furthermore, due to its lipophilic nature, BPA easily crosses the blood-brain and blood-placental barriers and has been identified in fetal blood, cord blood, breast milk, and amniotic fluid, with bioaccumulation in the maternal-fetal-placental unit [3,38,39,40,41].

Many manufacturers have begun to employ next-generation BPA replacement chemicals as a result of substantial scientific investigation, public awareness, and legislative restrictions. Several replacements, including bisphenol B (BPB), bisphenol F (BPF), bisphenol S (BPS), and bisphenol AF (BPAF), have now been developed and utilized in everyday products. The new “BPA-free” alternatives are all structurally very similar to the parent molecule BPA, with only minor additions or replacements of R groups on the original phenolic structure [3].

**Figure 1:**
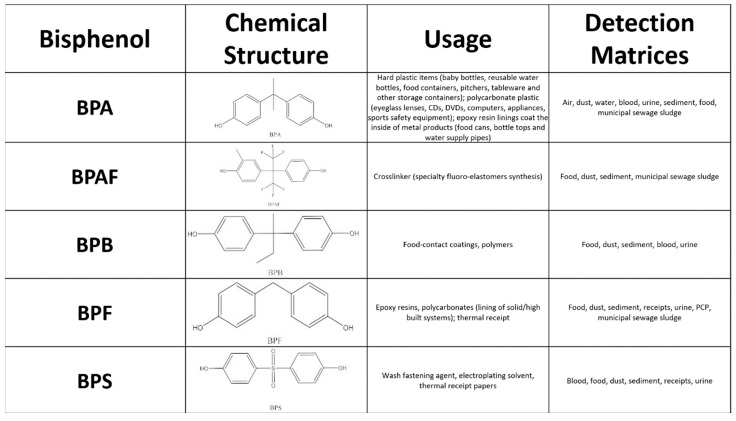
Chemical structure, usage, and detection matrices of BPA, BPAF, BPB, BPF, and BPS.

BPAF, BPB, BPF, and BPS remain to be investigated at the same level as BPA because the structural similarities between them are undeniable, as seen in Figure 1. With relatively limited toxicology and in vitro research, these next-generation chemicals must be investigated for complete toxicological characterization. Understanding how BPA analogs may alter cell viability and steroidogenesis is critical for characterizing the risk factors and hazards of these compounds, to which humans are increasingly exposed. Furthermore, little research has been conducted to investigate the effects of BPA analogs on mammalian or human cells. As a result, the purpose of this study was to investigate the effect of BPA, BPAF, BPB, BPF, and BPS at concentrations 0.05–100 µM on mitochondrial activity and steroidogenesis (intracellular cholesterol concentration, secretion of testosterone, progesterone, estradiol, and cortisol) in human adrenocortical carcinoma cells H295R in vitro.

## 2. Materials and Methods

### 2.1. H295R Cell Culture and Treatment

The NCI-H295R cells were obtained from the American Type Culture Collections (ATCC CRL-2128; ATCC, Manassas, VA, USA). The cells were cultured using protocols that had previously been established and validated. After the initiation of the H295R culture from the original ATCC batch, the H295R cells were cultured throughout three passages, then split and frozen in liquid nitrogen. Using H295R batches from frozen stocks, the cells used in the scheduled experiments were cultured for a minimum of three additional passages to achieve optimal hormone production. The H295R cells were grown in 25 cm^2^ plastic tissue culture flasks (TPP, Trasadingen, Switzerland) in Dulbecco’s Modified Eagle’s Medium/Nutrient F-12 Ham 1:1 mixture (Sigma, St. Louis, MO, USA) supplemented with 1.2 g/L NaHCO_3_ (Molar Chemicals, Halasztelek, Hungary), 12.5 mL/L of BD Nu-Serum (BD Bioscience, Bath, UK), and 5 mL/L of ITSC Premix (BD Bioscience) in a CO_2_ incubator at 37 °C with a 5% CO_2_ atmosphere. The culture medium was changed three times per week. With 0.25% trypsin-EDTA (Sigma-Aldrich, St. Louis, MO, USA) for 3 min, the H295R cells were detached from the bottom of the culture flasks. The cells were then centrifuged for 10 min at 125× *g* before being resuspended in a fresh cell culture medium. The TC20 automated cell counter (Bio-Rad, Oslo, Norway) was used to count the cells and adjust the concentration to the required level. The cell suspension was plated into sterile 96-well cell culture plates (6 × 10^4^ cells/100 µL/well) for mitochondrial activity and hormone measurements and into 6-well cell culture plates (1 × 10^6^ cells/2 mL/well). The cells were incubated for 24 h in a CO_2_ incubator at 37 °C under a humidified atmosphere of 95% air and 5% CO_2_ [42]. To explore the effect of bisphenols, cells were cultured for 24 h in a medium containing specific concentrations of each bisphenol (0.05, 0.1, 0.5, 1, 10, 25, 50, 75, 100 µM) (Sigma-Aldrich, St. Louis, MO, USA). Cells without any treatment were served as a control group (C), and DMSO (≤0.1%) was served as a negative control (NC).

### 2.2. Mitochondrial Activity Assay

The MTT (3–4,5-dimetyltiazol-2-yl)-2,5-diphenyltetrazolium bromide) (Sigma-Aldrich, St. Louis, MO, USA) assay, which measures the reduction of a yellow tetrazolium salt to insoluble blue formazan in the mitochondria of viable cells, was used to determine the mitochondrial activity of adrenocortical carcinoma cells exposed to different concentrations of bisphenols [43]. After 24 h of treatment, cells were incubated in a CO_2_ incubator for 1 h with MTT tetrazolium salt (Sigma-Aldrich, St. Louis, MO, USA). The supernatants were then removed, and the formed formazan crystals were dissolved with isopropanol (p.a. CentralChem, Bratislava, Slovak Republic). An ELISA reader (Multiscan FC, ThermoFisher Scientific, Vantaa, Finland) was used to measure dissolved formazan at 570 nm against 620 nm wavelengths. For each treatment, cells from four different experiments were examined. All of the data were expressed as a percentage of the control group [44].

### 2.3. CFDA-AM Assay

5-Carboxyfluorescein diacetate, Acetoxymethyl Ester (CFDA-AM) is a non-polar non-fluorescent dye capable of passing through the cell membrane. CFDA-AM (Invitrogen, CA, USA) is transformed by the hydrolytic activity of non-specific esterases into the fluorescent product carboxyfluorescein, which is also accumulated in the cytoplasm. The intensity of the conversion is an indicator of the plasma membrane, as long as the integrity of the membrane is not disturbed, a suitable environment for the esterase advantage. The amount of carboxyfluorescein is therefore dependent on the integrity of the cells, and in practice, the measurement of fluorescence at the excitation/emission wavelength of 485/520 nm was evaluated using GloMax^®^ Microplate Reader (Promega, Madison, WI, USA).

### 2.4. Assessment of Intracellular Cholesterol Concentration

A 24 h pre-cultivation of cells in 6-well plates was followed by a 24 h exposure to bisphenols at concentrations ranging from 0.05 to 100 µM. Intracellular cholesterol was measured in cell lysates using commercially available Randox kits (Randox Laboratories Ltd., Crumlin, UK) on a fully automated Rx Monaco biochemical analyzer (Randox Laboratories Ltd., Crumlin, UK). Individual experimental groups’ concentrations of CHOL were converted to % and compared to the concentration in the control group, while the concentration was set to 100%. 

### 2.5. Assessment of Steroid Hormone Production

After a 24 h culture in the presence of selected bisphenols concentrations, the medium was collected and centrifuged at 300× *g* for 10 min at 4 °C. The supernatant was stored at −20 °C. The enzyme-linked immunosorbent assay (ELISA) was used to determine steroid hormones directly from aliquots of the culture medium. The concentration of progesterone from the culture medium was evaluated by enzyme-linked immunosorbent assay using the ELISA kits (Cat. #K00225, Dialab, Austria) according to the instructional manual. The concentrations of estradiol were quantified using ELISA kits (Cat. #K00194, Dialab, Austria). The concentrations of testosterone were quantified using ELISA kits (Cat. #K00234, Dialab, Austria). The concentrations of cortisol were quantified using ELISA kits (Cat. #K00201, Dialab, Austria). The absorbance was measured at 450 nm by an ELISA reader (Multiscan FC, ThermoFisher Scientific, Vantaa, Finland). All of the data were expressed as a percentage of the control group.

### 2.6. Statistical Analysis

The collected data were statistically analyzed using GraphPad Prism 8 (GraphPad Software Incorporated, San Diego, CA, USA). The descriptive statistical parameters (minimum, maximum, and standard error) were initially evaluated. One-way analysis of variance (ANOVA) and Dunnett’s multiple comparison tests were employed to analyze differences between bisphenol treatments and the control. The significance levels were chosen at *** (*p*  <  0.001), ** (*p*  <  0.01), and * (*p*  <  0.05). Data were collected from six different sets of experiments (*n* = 6) with cells from different passages. The results were provided as means (±SEM) of mitochondrial activity %, cholesterol % progesterone %, testosterone %, estradiol %, and cortisol % cells of a control group, which represents 100%. 

## 3. Results

### 3.1. Viability Parameters

#### 3.1.1. Mitochondrial Activity

**Figure 2:**
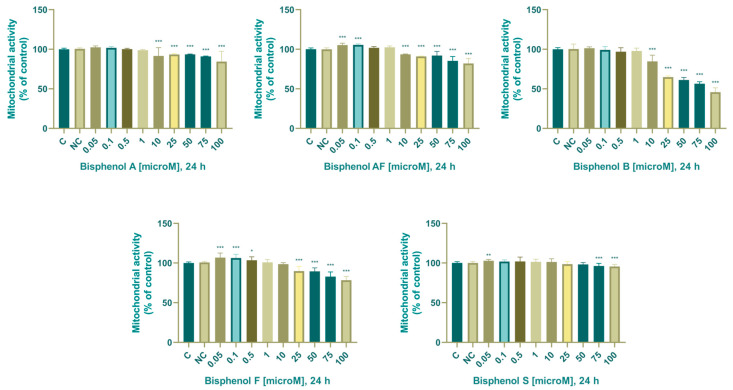
Effect of BPA, BPAF, BPB, BPF, and BPS on mitochondrial activity. Each bar indicates the mean value (±SEM) of the optical density as a percentage of the control group (C), which represents 100%, values are expressed as % of the control group. The level of statistical significance of the differences between the control and experimental groups was evaluated at the * *p* < 0.05, ** *p* < 0.01, and *** *p* < 0.001 levels of significance.

The initial increase in mitochondrial activity observed after 24 h of culture with BPA at concentrations of 0.05–0.5 µM was not at a statistically significant level. Although a dose of 1 µM of bisphenol A initiated a decrease in mitochondrial activity, we observed a significantly lower mitochondrial activity after 24 h cultivation with 10 (95.3 ± 6.51%), 25 (93.5 ± 1.09%), 50 (93.6 ± 0.53%), 75 (90.9 ± 0.53%), and 100 (85.1 ± 9.29%) µM. The effect of BPA on mitochondrial activity is shown in Figure 2.

The initial increase in mitochondrial activity observed after 24 h of culture at concentrations of 0.05 and 0.1 µM BPAF was at a statistically significant level: 0.05 µM (105 ± 2.33%) and 0.1 µM (106 ± 1.11%). We observed a significant decrease in mitochondrial activity after 24 cultures with 10 (91.3 ± 6.39%), 25 (89.4 ± 5.49%), 50 (92.1 ± 4.94%), 75 (85.4 ± 5.20%), and 100 (82.1 ± 6.21%) µM BPAF. The effect of BPAF on mitochondrial activity is shown in Figure 2.

The initial increase in mitochondrial activity observed after 24 of culture at 0.05 µM BPB concentration was statistically nonsignificant. The results of the analysis show that as the BPB concentration increases, the mitochondrial activity of the cells is disrupted. After 24 h, we noted a statistically significant decrease after cultivation with 10–100 µM (10 (84.7 ± 7.60%), 25 (64.1 ± 4.47%), 50 (59.7 ± 5.99%), 75 (55.7 ± 4.15%), and 100 (45.1 ± 5.95%) µM). The effect of BPB on mitochondrial activity is shown in Figure 2.

The initial increase in mitochondrial activity after 24 h of culture at a concentration of 0.05–0.5 µM was statistically significant (0.05 µM = 107 ± 5.50%, 0.1 µM = 106 ± 4.48%, 0.5 µM = 103 ± 4.25%). The results of the analysis show that as the BPF concentration increases, the mitochondrial activity of the cells is more disturbed. After 24 h, we observed a statistically significant decrease in mitochondrial activity after cultivation with 25, 50, 75, and 100 µM (25 (89.8 ± 5.51%), 50 (89.5 ± 4.16%), 75 (82.8 ± 5.74%), and 100 (78.3 ± 4.60%) µM). The effect of BPF on mitochondrial activity is shown in Figure 2.

Using the MTT test, we demonstrated that the mitochondrial activity of H295R cells after 24 h cultivation with 0.05 µM BPS significantly increased (103 ± 1.75%) and significantly decreased after cultivation with 75 and 100 µM BPS (75: 96.2 ± 3.19%, 100: 95.5 ± 2.56%). The effect of BPS on mitochondrial activity is shown in Figure 2.

#### 3.1.2. Membrane Integrity

Another parameter of the cellular structures we monitored was the integrity of the cell membrane determined using carboxyfluorescein diacetate (CFDA-AM). After 24 h of cultivation, we noted a significant increase at 0.05 µM (102 ± 1.76%) and a significant decrease at 10 (96.2 ± 1.67%), 25 (92.6 ± 1.09%), 50 (92.7 ± 0.53%), 75 (89.7 ± 0.53%), and 100 (84.9 ± 3.79%) µM. From these results, it follows that bisphenol A affected the integrity of the cell membrane more significantly than the mitochondrial activity, as we recorded a significant decrease in the integrity of the cell membrane already at a concentration of 10 µM in both cultivation times.

After a 24 h culture of adrenocarcinoma cells with BPAF, we observed a significant increase after culture with 0.05 (105 ± 2.33%) and 0.1 (105 ± 1.11%) µM. Furthermore, at concentrations of 10–100 µM, we noted a significant decrease in cell membrane integrity (24 h: 10 (92.6 ± 0.53%), 25 (90 ± 0.53%), 50 (91.2 ± 4.94%), 75 (84.1 ± 5.20%), and 100 (80.5 ± 6.21%) µM).

After 24 h of exposure to BPB, we noted a significant decrease in cell membrane integrity in experimental groups exposed to 0.5 and 10–100 µM (0.5 (96 ± 4.72%), 10 (82.7 ± 7.60%), 25 (63 ± 1.42%), 50 (59.4 ± 2.96%), 75 (54 ± 2.37%), and 100 (42.6 ± 5.20%) µM).

BPF in concentrations of 0.05 and 0.1 µM significantly increased the integrity of the cell membrane (24 h: 0.05 (106 ± 5.50%), 0.1 (106 ± 4.48%) µM. A significant decrease in the monitored parameter occurred after cultivation with 25–100 µM BPF: (24 h: 25 (88.9 ± 5.51%), 50 (88.6 ± 4.16%), 75 (81.5 ± 5.74%), and 100 (76.9 ± 4.60%) µM).

We noted a significant decrease in the integrity of the cellular membrane compared to the control in the following experimental groups: (24 h: 50 µM (97.2 ± 2.31%), 75 µM (95 ± 3.19%), and 100 µM (93.9 ± 2.56%)).

**Figure 3:**
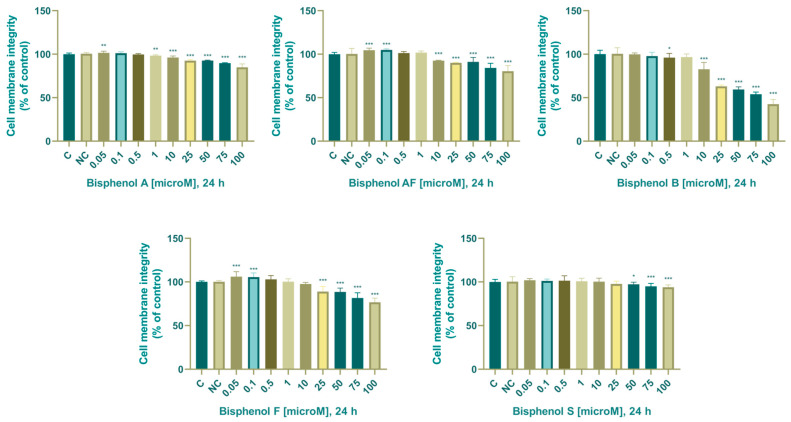
Effect of BPA, BPAF, BPB, BPF, and BPS on cell membrane integrity. Each bar indicates the mean value (±SEM) of the optical density as a percentage of the control group (C), which represents 100%, values are expressed as % of the control group. The level of statistical significance of the differences between the control and experimental groups was evaluated at the * *p* < 0.05, ** *p* < 0.01, and *** *p* < 0.001 levels of significance.

The effect of BPA, BPAF, BPB, BPF, and BPS on the integrity of the cellular membrane is shown in Figure 3.

### 3.2. Steroidogenesis

#### 3.2.1. Cholesterol

In the control samples, the intracellular concentration of cholesterol was 0.113 ± 0.001 mmol/g proteins (24 h). After 24 h, we noted significant changes in the concentration of intracellular cholesterol in experimental groups cultivated with 0.05 (83.9 ± 3.82%), 0.1 (78.3 ± 4.62%), 0.5 (72.3 ± 1.18%), 1 (77.5 ± 3.95%), 25 (120.6 ± 5.26%), 50 (137.9 ± 4.40%), 75 (157.4 ± 4.20%), and 100 (168.7 ± 9.60%) µM. BPA.

In the control samples, the intracellular concentration of cholesterol was 0.117±0.001 mmol/g proteins. After 24 h cultivation, we noted significant changes in experimental groups cultured with 0.1 (81.8 ± 1.69%), 0.5 (77.02 ± 3.76%), 1 (76.4 ± 3, 57%), 10 (74.0 ± 0.94%), 25 (65.7 ± 7.04%), 50 (72.7 ± 2.40%), 75 (84.2 ± 2.37%), and 100 (111.5 ± 3.80%) µM BPAF.

In the control samples, the intracellular concentration of cholesterol was 0.104±0.001 mmol/g of proteins (24 h). When determining the effect of BPB on cholesterol, we established a similar trend of changes for BPA and BPAF. After 24 h of cultivation, we noted a significant decrease after cultivation with 1 µM (89.5 ± 0.59%) and a significant increase after cultivation with 10 (116.7 ± 2.85%), 25 (149.1 ± 4.36 %), 50 (165.5 ± 3.11%), 75 (169.8 ± 0.90%), and 100 (157.1 ± 5.99%) µM BPB.

In the control samples, the intracellular concentration of cholesterol was 0.119 ± 0.002 mmol/g of proteins (24 h). After 24 h, we noted a statistically significant decrease at concentrations of 0.5–75 µM (0.5 µM = 90.6 ± 0.71%; 1 µM = 87.6 ± 1.49%; 10 µM = 81.8 ± 1.69%; 25 µM = 77.0 ± 3.76%; 50 µM = 80.47 ± 1.48%; 75 µM = 84.0 ± 0.58%;), and a statistically significant increase after cultivation with 100 µM (130 ± 4.55%) µM. 

In the control samples, the intracellular concentration of cholesterol was 0.095 ± 0.001 mmol/g proteins (24 h). When determining the effect of BPS on cholesterol, we noted significant changes in all experimental groups. After 24 h of cultivation, we noted a significant decrease after cultivation with 0.05–10 µM and a significant increase after cultivation with 25–100 µM: 0.05 µM (93.8 ± 1.05%), 0.1 µM (94.1 ± 1.70%), 0.5 µM (90.8 ± 0.37%), 1 µM (86.2 ± 0.57%), 10 µM (84.1 ± 1.87%), 25 µM (115.1 ± 2.85%), 50 µM (121.4 ± 2.35%), 75 µM (125.7 ± 2.41%), and 100 µM (139.1 ± 1.45%). 

**Figure 4:**
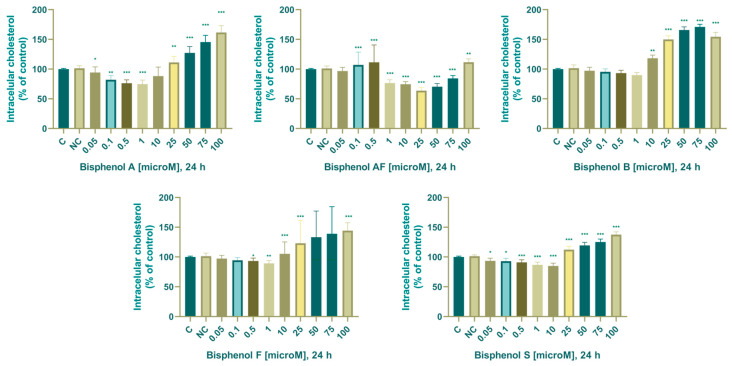
Effect of BPA, BPAF, BPB, BPF, and BPS on intracellular cholesterol concentration. Each bar indicates the mean value (±SEM) of the optical density as a percentage of the control group (C), which represents 100%, values are expressed as % of the control group. The level of statistical significance of the differences between the control and experimental groups was evaluated at the * *p* < 0.05, ** *p* < 0.01, and *** *p* < 0.001 levels of significance.

The effect of BPA, BPAF, BPB, BPF, and BPS on concentration of progesterone is shown in Figure 4.

#### 3.2.2. Progesterone

In the control samples, the concentration of progesterone was 20.041 ± 0.249 ng/mL (24 h). Progesterone production was affected by bisphenol A biphasically. We noted a significant increase in progesterone secretion after 24 h of cultivation with 0.05 (105 ± 4.49%), 0.1 (106 ± 4.08%), and 0.5 (103 ± 4.49%) µM; we noted a decrease after cultivation with 10 (91 ± 4.61%), 25 (84.6 ± 4.27%), 50 (81.2 ± 4.61%), 75 (76 ± 4.61%), and 100 (71.3 ± 4.53%) µM. BPA.

In the control samples, the concentration of progesterone was 19.893 ± 0.308 ng/mL. To determine the concentration of progesterone, adrenocarcinoma cells were cultured with the addition of bisphenol AF for 24 h under in vitro conditions. The results of the analysis demonstrated that concentrations of 0.05–50 µM induced a significant increase and a concentration of 100 µM induced a significant decrease in the secretion of detectable progesterone. Changes in progesterone concentration after 24 h exposure were as follows: 0.05 (104 ± 4.49%), 0.1 (104 ± 4.74%), 0.5 (108 ± 4.65%), 1 (110 ± 4.74%), 10 (121 ± 4.55%), 25 (135 ± 3.87%), 50 (120 ± 4.90%), 100 (93.7 ± 4.10%) µM BPAF. 

In the control samples, the concentration of progesterone was 20.035 ± 0.395 ng/mL (24 h). To determine progesterone concentration, adrenocarcinoma cells were cultured with the addition of BPB for 24 h under in vitro conditions. The results of the analysis demonstrated that concentrations of 0.05 and 0.1 µM induced an increase and concentrations of 1–100 µM induced a decrease in the secretion of detectable progesterone. Significant changes in progesterone concentration after 24 h exposure were as follows: 0.1 (104 ± 4.42%), 10 (86.1 ± 4.61%), 25 (57.6 ± 4.63%), 50 (54.4 ± 4.61%), 75 (47.7 ±4.61%), 100 (42.7 ± 4.63%) µM. 

In the control samples, the concentration of progesterone was 20.156 ± 0.250 ng/mL. To determine progesterone concentration, adrenocarcinoma cells were cultured with the addition of BPF for 24 h under in vitro conditions. Quantification of progesterone from an aliquot of the culture medium demonstrated that concentrations of 0.05–75 µM induced an increase and a concentration of 100 µM induced a decrease in the secretion of detectable progesterone. Significant changes in progesterone concentration after 24 h exposure were as follows: 0.05 (105 ± 4.39%), 0.1 (104 ± 4.42%), 0.5 (105 ± 4.08%), 1 (107 ± 4.39%), 10 (115 ± 5.81%), 25 (121 ± 4.60%), 50 (136 ± 4.60%), 75 (126 ± 4.60%), 100 (90 ± 5.03%) µM. 

In the control samples, the concentration of progesterone was 19.948 ± 0.309 ng/mL (24 h). To determine progesterone concentration, adrenocarcinoma cells were cultured with the addition of BPS for 24 h under in vitro conditions. Quantification of progesterone from an aliquot of culture medium demonstrated that concentrations of 0.05–10 µM induced an increase and concentrations of 25–100 µM induced a decrease in detectable progesterone secretion. Significant changes in progesterone concentration after 24 h exposure were as follows: 0.05 (104 ± 3.87%), 0.1 (107 ± 4.60%), 0.5 (110 ± 4.55%), 1 (111 ± 4.39%), 10 (117 ± 5.67%), 25 (89.7 ± 5.08%), 50 (85.1 ± 4.53%), 75 (70 ± 4.27%), 100 (61.5 ± 4.61%) µM.

**Figure 5:**
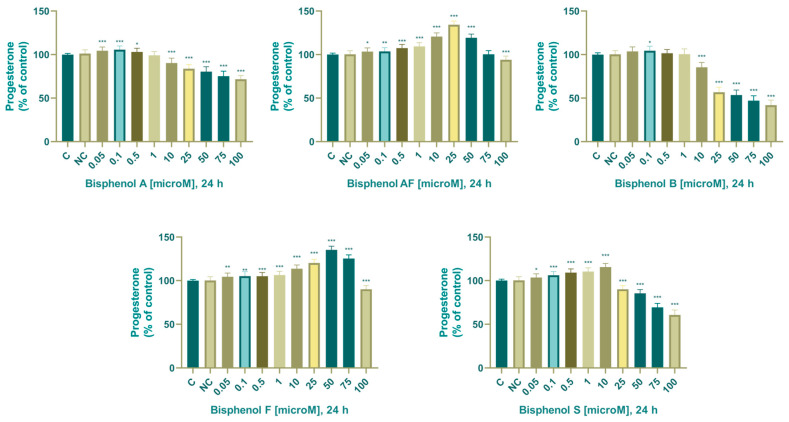
Effect of BPA, BPAF, BPB, BPF, and BPS on progesterone production. Each bar indicates the mean value (±SEM) of the optical density as a percentage of the control group (C), which represents 100%, values are expressed as % of the control group. The level of statistical significance of the differences between the control and experimental groups was evaluated at the * *p* < 0.05, ** *p* < 0.01, and *** *p* < 0.001 levels of significance.

The effect of BPA, BPAF, BPB, BPF, and BPS on concentration of progesterone is shown in Figure 5.

#### 3.2.3. Estradiol

In the control samples, the concentration of estradiol was 1.017 ± 0.013 ng/mL (24 h). After 24 h of exposure, we noticed a significant effect (increase: 1–25 µM; decrease: 50–100 µM) on estradiol production in the experimental groups: 1 (105 ± 4.08%), 10 (107 ± 4.25%), 25 (110 ± 4.15%), 50 (94.8 ± 5.64%), 75 (89.7 ± 5.88%), and 100 (83.6 ± 6.26%) µM. 

In the control samples, the concentration of estradiol was 0.985 ± 0.015 ng/mL. After 24 h of exposure, we noted significant changes (increase: 0.05–10 µM; decrease: 50-100 µM) in estradiol production in the experimental groups: 0.05 (102 ± 6.83%), 0.1 (105 ± 5.31%), 0.5 (105 ± 4.32%), 10 (108 ± 4.74%), 50 (95.2 ± 4.53%), 75 (92.8 ± 4.69%), and 100 (87.5 ± 6.92%) µM. 

In the control samples, the concentration of estradiol was 1.018 ± 0.042 ng/mL. After 24 h of exposure, we noted a significant effect (increase: 0.05, 0.1 µM; decrease: 10–100 µM) on estradiol production in the experimental groups: 0.05 (104 ± 4.05%), 0.1 (107 ± 4.10%), 10 (92.8 ± 4.15%), 25 (64.7 ± 3.94%), 50 (61.1 ± 4.12%), 75 (56.4 ± 4.32%), and 100 (51 ± 4.26%) µM. 

In the control samples, the concentration of estradiol was 0.992 ± 0.012 ng/mL. After 24 h of exposure, we noticed a significant increase in estradiol production in the experimental groups: 10 (104 ± 4.11%), 25 (120 ± 4.30%), 50 (125 ± 4.09%), 75 (122 ± 4.18%) µM, and significant decrease in the experimental group 100 (96.6 ± 3.97%) µM. 

In the control samples, the concentration of estradiol was 1.014 ± 0.016 ng/mL. After 24 h of exposure, we noted a significant influence (decrease: 0.05–75 µM; increase: 100 µM) on estradiol production in the experimental samples: 0.05 (95.4 ± 4.08%), 0.1 (95.9 ± 4.11%), 0.5 (94.4 ± 4.10%), 1 (96.6 ± 4.32%), 10 (106 ± 4.23%), 25 (90 ± 3.89%), 50 (88.4 ± 4.39%), 75 (88.9 ± 4.38%), and 100 (81.2 ± 5.03%) µM. 

**Figure 6:**
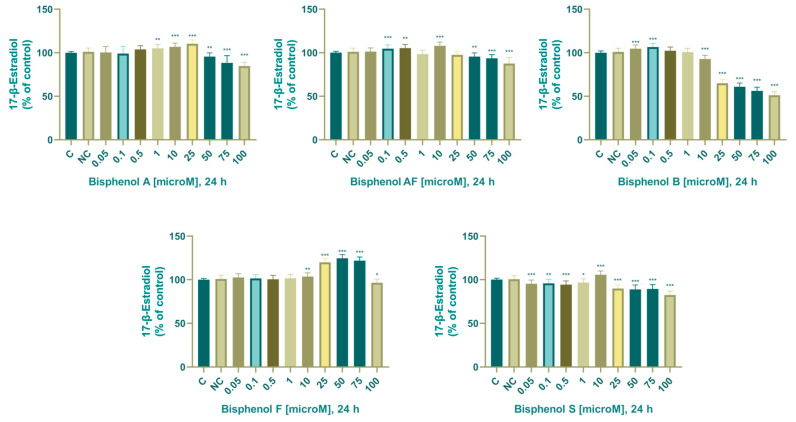
Effect of BPA, BPAF, BPB, BPF, and BPS on estradiol production. Each bar indicates the mean value (±SEM) of the optical density as a percentage of the control group (C), which represents 100%, values are expressed as % of the control group. The level of statistical significance of the differences between the control and experimental groups was evaluated at the * *p* < 0.05, ** *p* < 0.01, and *** *p* < 0.001 levels of significance.

The effect of BPA, BPAF, BPB, BPF, and BPS on concentration of estradiol is shown in Figure 6.

#### 3.2.4. Testosterone

In the control samples, the concentration of testosterone was 9.851 ± 0.122 ng/mL (24 h). After 24 h exposure of adrenocarcinoma cells to BPA, we noted a significant decrease in testosterone production in experimental groups cultured with 0.1 (91.8 ± 5.87%), 0.5 (90.2 ± 4.63%), 1 (85.9 ± 8.36%), 10 (75.8 ± 4.63%), 25 (67 ± 6.18%), 50 (58.2 ± 8.92%), 75 (53 ± 8.95%), and 100 (45.9 ± 11.40%) µM.

In the control samples, the concentration of testosterone was 10.047 ± 0.156 ng/mL. After 24 h exposure of adrenocarcinoma cells to BPAF, we noted a significant decrease in testosterone production in experimental groups cultured with: (24 h: 0.05 (94.6 ± 4.39%), 0.1 (93.5 ± 4.60%), 0.5 (93.2 ± 3.87%), 1 (90.4 ± 4.61%), 25 (96 ± 9.51%), 50 (72.7 ± 3.87%), 75 (68.8 ± 5.08%), and 100 (60.7 ± 4.53%) µM). 

In the control samples, the concentration of testosterone was 10.256 ± 0.202 ng/mL. After a 24 h exposure of adrenocarcinoma cells to BPB, we noted a significant decrease in testosterone production in experimental groups cultured with 0.1 (94.5 ± 4.71%), 0.5 (92.2 ± 4.39%), 1 (92.4 ± 4.63%), 10 (78.9 ± 4.27%), 25 (57.6 ± 4.27%), 50 (57.1 ± 4.42%), 75 (51 ± 3.79%) and 100 (39.3 ± 5.47%) µM. 

In the control samples, the concentration of testosterone was 9.769 ± 0.121 ng/mL. After 24 h exposure of adrenocarcinoma cells to BPF, we noted a significant decrease in testosterone production in experimental groups cultivated with: (24 h: 1 (95.1 ± 4.08%), 10 (90.3 ± 5.08%), 25 (82.8 ± 5.99%), 50 (78.8 ± 5.08%), 75 (71.7 ± 3.96%) and 100 (67.4 ± 5.80%) µM). 

The concentration of testosterone in the samples was 10.027 ± 0.155 ng/mL. After a 24 h exposure of adrenocarcinoma cells to bisphenol S, we noted a significant decrease in testosterone production in experimental histories cultured with: (0.05 (82.7 ± 3.87 %), 0.1 (80.7 ± 6.50 ), 0.5 (80.5 ± 4.49%), 1 (76.4 ± 4.08%), 10 (95.2 ± 4.53%), 25 (58.2 ± 5.64%), 50 (52.7 ± 3.96%), 75 (45.3 ± 5.76%) and 100 (40.7 ± 5.08%) µM). 

**Figure 7:**
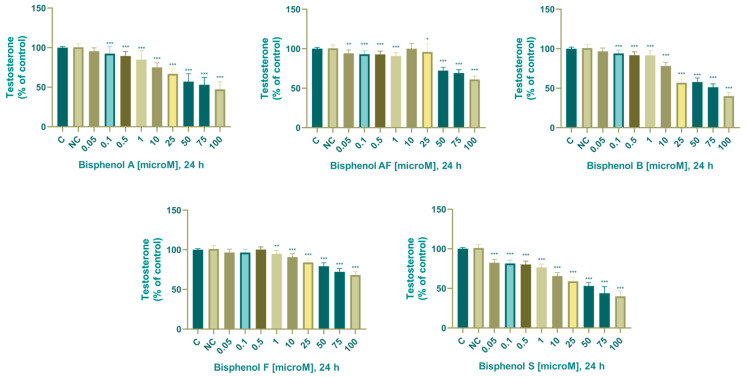
Effect of BPA, BPAF, BPB, BPF, and BPS on testosterone production. Each bar indicates the mean value (±SEM) of the optical density as a percentage of the control group (C), which represents 100%, values are expressed as % of the control group. The level of statistical significance of the differences between the control and experimental groups was evaluated at the * *p* < 0.05, ** *p* < 0.01, and *** *p* < 0.001 levels of significance.

The effect of BPA, BPAF, BPB, BPF, and BPS on concentration of testosterone is shown in Figure 7.

#### 3.2.5. Cortisol

In the control samples, the concentration of cortisol was 7.572 ± 0.094 ng/mL (24 h). Bisphenol A significantly affected the secretion of cortisol after 24 h of exposure; however, unlike the secretion of progesterone, we did not observe a biphasic effect. Cortisol production after 24 h of exposure decreased compared to the control group in the following experimental groups: 0.1 (95.2 ± 5.49%), 0.5 (92 ± 4.39%), 1 (88 ± 4.10 %), 10 (89.5 ± 4.59%), 25 (63.6 ± 4.10%), 50 (59.1 ± 4.40%), 75 (52.2 ± 4.43%), and 100 (46.6 ± 7.17%) µM. 

In the control samples, the concentration of cortisol was 8.046 ± 0.125 ng/mL. Bisphenol AF significantly affected the secretion of cortisol after 24 h of exposure and, as with the secretion of progesterone, we noted a biphasic effect. Cortisol production after 24 h of exposure was as follows: 0.05 (107 ± 4.90%), 0.1 (109 ± 5.75%), 1 (90.7 ± 4.90%), 10 (87.9 ± 4.10%), 25 (71.3 ± 4.10%), 50 (65.2 ± 4.10%), 75 (60 ± 4.10%), and 100 (55.9 ± 4.10 %) µM. Cortisol production increased compared to the control group in the following experimental groups: 0.05 (107 ± 4.90%), 0.1 (109 ± 5.75%) µM. Cortisol production decreased compared to the control group in the following experimental groups: 1 (90.7 ± 4.90%), 10 (87.9 ± 4.10%), 25 (71.3 ± 4.10%), 50 (65.2 ± 4.10%), 75 (60 ± 4.10%), and 100 (55.9 ± 4.10 %) µM.

In the control samples, the concentration of cortisol was 7.509 ± 0.148 ng/mL. BPB significantly affected cortisol secretion after 24 h exposure and, as with progesterone secretion, we noted a biphasic effect (increase up to a concentration of 0.5 µM and decrease from a concentration of 1 µM BPB). Significant decrease in cortisol production after 24 h of exposure were as follows: 10 (90 ± 4.27%), 25 (75 ± 6.27%), 50 (69.3 ± 4.91%), 75 (60.6 ± 4.91%), and 100 (51.7 ± 4.69%) µM. 

In the control samples, the concentration of cortisol was 7.864 ± 0.098 ng/mL. By quantifying cortisol from an aliquot amount of culture medium, we noted that bisphenol F significantly affected cortisol secretion after 24 h of exposure, and unlike progesterone secretion, where we noted a biphasic effect, we noted a gradual decrease in cortisol concentration with increasing exposure dose. Significant decrease in cortisol production after 24 h of exposure was as follows: 0.05 (95 ± 4.49%), 0.1 (92.6 ± 4.69%), 0.5 (93.4 ± 4.24%), 1 (96 ± 4.42%), 10 (91.1 ± 4.42%), 25 (89.9 ± 4.42%), 50 (89.2 ± 4.93%), 75 (82.3 ± 4.08%), 100 (78.6 ± 5.47%) µM. 

**Figure 8:**
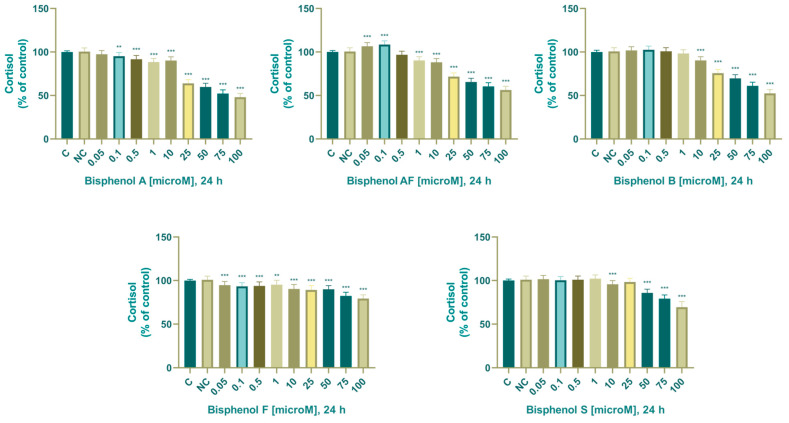
Effect of BPA, BPAF, BPB, BPF, and BPS on cortisol production. Each bar indicates the mean value (±SEM) of the optical density as a percentage of the control group (C), which represents 100%, values are expressed as % of the control group. The level of statistical significance of the differences between the control and experimental groups was evaluated at the ** *p* < 0.01, and *** *p* < 0.001 levels of significance.

The effect of BPA, BPAF, BPB, BPF, and BPS on concentration of cortisol is shown in Figure 8.

## 4. Discussion

Since the beginning of the Industrial Revolution, it has been observed that chemical production has been focused on increasing urbanization and industrial development. Because of the increased demand, the manufacturing of chemical goods such as insecticides, detergents, disinfectants, plastics, medicines, and personal care items has increased [45]. The usage of bisphenol A, a prevalent industrial chemical ingredient in numerous items, has gradually increased over the last 58 years. BPA production began commercially in the United States in 1957 and in Europe a year later. Global production growth is between 0% and 5% per year, with the recent increase in China, where the BPA market increased by an average of 13% between 2000 and 2006 [5]. A meta-analysis from 2021 confirms the significant prevalence of BPA in human samples, with the findings confirming the presence of BPA in more than 90% of samples [46,47]. Research published in 2020 compared a commonly used method for determining the levels of BPA in human samples to a new improved direct approach combining liquid chromatography and mass spectrometry. Using this direct technique, they discovered a geometric mean BPA in urine samples that were 44 times greater than the previously published geometric mean for US adults based on the National Health and Nutrition Examination Survey [48]. There are fewer studies examining the occurrence and concentration of bisphenol A alternatives; however, the presence of BPS in human tissues and body fluids has been shown without question [49,50,51,52,53,54,55,56,57]. The presence of BPB [58], BPAF [59,60,61], and BPF [54,55,56,57] have also been confirmed in human samples.

BPA has become one of the most studied endocrine disruptors, which has resulted in its legal restriction and replacement with equivalents such as BPAF, BPB, BPF, and BPS. Because of their similar chemical properties, bisphenol AF, B, F, and S are widely industrially utilized as BPA alternatives, raising concerns about their biological implications. Environmental monitoring studies reveal that BPA is losing its environmental dominance, while other bisphenols, particularly BPS, BPF, and BPAF, are becoming more prevalent [62]. The health risks associated with bisphenol A analogs need to be thoroughly studied, given the fact that a comparable or greater negative effect is predicted due to the chemical structural similarities with BPA [54,63,64,65].

The concentrations of bisphenols used in this article were chosen based on previous research [42,44], but also on the publications of other researchers [29,66,67]. Since concentrations of 1–10 μΜ, BPA is comparable to the range of detected levels in the blood serum of occupationally exposed individuals [68], we investigated the effect of bisphenols in this range in five experimental groups—0.05 µM, 0.1 µM, 0.5 µM, 1 µM, and 10 µM, and for a more comprehensive evaluation of the activity of bisphenols in a larger concentration range due to the constantly increasing concentration of bisphenols and the environment, we added higher concentrations of bisphenols, namely 25 µM, 50 µM, 75 µM, and 100 µM.

### 4.1. BPA Alternatives Affected Cell Viability

Mitochondria are a very important cell organelle ensuring the course of essential processes such as oxidative phosphorylation, the Krebs cycle, and other processes of energy acquisition. They are of particular importance in the cells of the adrenal glands—they are responsible for the process of steroid hormone formation. Important sub-reactions of steroidogenesis take place in the mitochondria. Endocrine disruptors can inhibit the activity of mitochondria [69], which are also the preferred target of many toxicants [70]. In the presented article, we therefore decided to determine the effect of bisphenols on the mitochondrial activity of adrenocarcinoma cells, the damage of which could negatively affect their secretory activity. Based on the MTT test results, we may conclude that BPB is the most toxic bisphenol since it significantly reduces mitochondrial activity at a concentration of 25 µM. Even at the highest concentration, BPS demonstrated the least toxicity, with mitochondrial activity remaining at over 88%, and BPA with mitochondrial activity remaining above 80% at the highest concentration. Mitochondrial toxicity of the five bisphenols according to our experiments was BPB > BPF > BPAF > BPA > BPS. The most prominent biphasic effect was observed after BPF and BPAF exposure since low concentrations of these endocrine disruptors enhanced mitochondrial function, most likely by cell proliferation stimulation. The high cytotoxicity of BPB was also confirmed by another study, the results of which indicate that in H295R cells BPB showed cytotoxicity at 25 µM, the other four bisphenol compounds including BPA, BPAF, BPF, and BPS did not show cytotoxicity at the given concentration [67]. Since viability below 80% can be considered cytotoxic, the results of our experiments are consistent with the results of this study published in 2021. Our findings are supported by another study that determined the toxicity of BPA, BPF, and BPS on the H295R cell line and found that toxicity was not observed at experimental doses up to 30 µM [71]. Feng et al. (2012) determined the viability of H295R cells exposed to 10–500 µM BPF, BPA, BPS, and BPAF for 24 h by CCK-8 assay. The results of their study indicated that toxicity increased with increased exposure concentration of BPs. BPAF had the greatest toxicity with 16.1% cell viability at 200 µM for 24 h; followed by BPA with 60.2% cell viability at 200 µM for 24 h. BPS was less toxic than BPA, the percent cell viability was 62.6%-in the 200 µM BPS treatment group for 24 h. The least toxic bisphenol according to their study was BPF, which contradicts the results of our experiments [66]. Cho et al. (2018) evaluated viability in human endometrial stromal cells using MTT assay after cultivation with 1–100,000 pM BPA for 24. According to their research, none of the BPA concentrations tested were toxic for the cultured endometrial stromal cell [72]. These results are consistent with our results as mitochondrial activity remained above 80% after cultivation with the highest dose (100 µM BPA).

Based on the 5-CFDA-AM assay, we can conclude that the most cytotoxic bisphenol is BPB, as already at a concentration of 10 µM it showed a significant reduction in cell membrane integrity. The toxicity of the bisphenols was in the order BPB > BPF > BPAF > BPA > BPS. The most pronounced biphasic effect was recorded with BPAF, as its low concentrations stimulated the integrity of lysosomes and cell membranes, probably through stimulation of cell proliferation. The biphasic effect of bisphenols on viability was also detected in a 2017 study: Lan et al. (2017) demonstrated a concentration-dependent biphasic effect of bisphenol A in an in vitro study with MA 10 cells (a mouse Leydig cell tumor-derived model cell line). MA-10 cells were cultured with different concentrations of BPA (0.01 to 200 µM). Their research results show that low concentrations of BPA (0.01 to 0.1 µM) increase cell viability, while higher concentrations (100 to 200 µM) decrease cell viability [73]. Significant cytotoxicity of BPB is also described by Lin et al. (2021), in their in vitro study, they monitored the endocrine-disruptive potential of BPA analogs, in the first step of their experiments, before determining the effects on steroidogenesis, as in the experiments in our dissertation, they determined the effect of bisphenol A analogs on cellular parameters and cell viability. During their experiments, they focused on a defined concentration range of bisphenols, which was 1.56 to 25 µM. Statistically significant cytotoxicity was observed only after cultivation with 25 µM BPB. No significant level of cytotoxicity was observed at any of the concentrations of BPA, BPAF, BPF, and BPS used [67]. The results of their study are in line with the results of our experiments, as only bisphenol B showed cytotoxicity and an effect on damage to cellular structures (mitochondria, cell membrane) at a bisphenol concentration of 25 µM. Cytotoxicity of bisphenols is also reported in another study on stem cells. The results of experiments performed on rats and human stem cells underline the toxicity and biological efficacy of BPA analogs and establish the order of biological effect: BPAF > BPA > BPS [3]. The order of cytotoxicity of bisphenols determined by these experiments is in agreement with our results, as we observed cytotoxicity in the order BPAF > BPA > BPS. While the mechanism of BPA, BPAF, BPB, BPF, and BPS cytotoxicity was not addressed beyond mitochondrial activity and membrane integrity in this study, several prior studies have shown that BPA is capable of inducing DNA damage in a variety of cell types at high micromolar concentrations [68,74].

### 4.2. BPA Alternatives Impaired Steroidogenesis

Endocrine disruption is toxicity with both physiological and regulatory implications; steroid hormones regulate reproduction, development, and other biological processes, and identifying substances that may interfere with hormone synthesis is a prime concern [75]. In this study, we investigated whether BPAF, BPB, BPF, and BPS are safe alternatives to BPA.

### 4.3. Intracellular Cholesterol

Cholesterol is the substrate essential for the enzyme CYP450scc to complete its cleavage and catalyze the conversion from cholesterol to pregnenolone [30]. There is a minimal number of studies aimed at determining the effect of bisphenols on intracellular cholesterol concentration in cell or animal models, and according to the PubMed database, no study has been performed in the cell model we used. Li et al. (2019) reported that BPA significantly increased the intracellular cholesterol concentration of HepG2 cells at concentrations of 1 nM and 10 nM and significantly decreased intracellular cholesterol concentration at concentrations of 100 nM, 1 µM, and 10 µM. The results of these authors indicate a biphasic effect of BPA on intracellular cholesterol concentration. They concluded that blocking SREBP2 (sterol regulatory element-binding protein could prevent the effect of BPA on intracellular cholesterol concentration [76]). The results of their study are partially consistent with our results, as we noted a significant decrease in cholesterol concentration after cultivation with 1 µM BPA, a significant increase in cholesterol in nanomolar concentrations of BPA cannot be compared with our results since the lowest concentration we used was 0.05 µM. Li et al. (2019) noted a decrease in cholesterol concentration even after cultivation with 10 µM BPA, which contradicts our results, as we observed an increase in cholesterol concentration with BPA and BPB and a decrease with BPAF, BPF, and BPS. We cannot compare concentrations higher than 10 µM, as Li et al. (2019) did not determine the effect of these concentrations on changes in cholesterol concentration. A meta-analysis of human samples from 2003 to 2014 revealed no significant associations between pediatric and adult urinary BPA concentrations, LDL cholesterol, and HDL cholesterol. However, the authors of this study state that due to the cross-sectional nature of the study, the results should be thoroughly clarified by longitudinal cohort studies with repeated measurements of BPA [48]. The results of another study on human samples suggest the involvement of BPA in cholesterol homeostasis, as they report that BPA is correlated with a higher prevalence of HDL-hypocholesterolemia [77]. According to the findings of another human investigation with 1872 individuals, higher levels of BPA were related to higher levels of serum LDL cholesterol, non-HDL cholesterol, and lower levels of HDL cholesterol in middle-aged and elderly Chinese adults [78]. Another research conducted in 2022 determined that PPARα activation disrupted homeostasis of cholesterol/testosterone after exposure to BPS, BPF, and BPAF [79]. Another study proposes that BPA disrupts cholesterol homeostasis in rat granulosa cells by decreasing the expression of a gene involved in the reverse cholesterol transport (Abca1) and increasing the level of proteins involved in the cholesterol transport (StAR) and biosynthesis (SREBP-1) [80]. We propose that BPA, BPAF, BPB, BPF, and BPS disturb cholesterol homeostasis in H295R human adrenocarcinoma cells based on the findings of our in vitro experiments and comparison with the literature. 

### 4.4. Progesterone

Following the application of bisphenol A, AF, B, F, and S, we obtained results confirming the sensitive response of cells to the presence of these chemicals via changes in signaling pathways linked with progesterone production and release. Progesterone is the first hormone generated in the steroidogenesis process because pregnenolone is regarded as a precursor of steroid hormones and is created from cholesterol by the action of cytochrome P450scc in the mitochondria. It is converted from pregnenolone by the 3β-HSD enzyme in the smooth endoplasmic reticulum [81]. Because we found a significant effect of all of the BPA analogs we tested and BPA itself on the production of progesterone by human adrenocarcinoma cells, we believe that bisphenols disrupt these enzymes.

Rosenmai et al. (2014) reported an increase in progesterone synthesis by H295R human adrenocarcinoma cells following BPF and BPS exposure but no significant changes after BPA and BPB exposure. They demonstrated an increase in 17-OH progesterone synthesis following exposure to BPB, BPF, and BPS, but no significant alterations after exposure to BPA [82]. BPS caused the greatest changes in 17-OH progesterone concentrations, whereas BPF caused the greatest changes in progesterone concentrations. In the published study, they revealed the influence of maximum effective doses on steroidogenesis ranging from 0.3 to 28 µM. Their findings are consistent with our findings because BPA and BPB had the least stimulating impact on progesterone synthesis in our experiments, whereas BPF and BPS stimulated progesterone production most significantly. We also examined the effects of BPAF, which had a stimulatory effect comparable to bisphenol F. Rosenmai et al. (2014) did not investigate this structural equivalent of bisphenol A. Bujnakova Mlynarcikova et al. (2021) found no significant alterations in progesterone production in pig granulosa cells following exposure to bisphenol A, AF, and F for 24, 48, and 75 h at concentrations ranging from 10^−9^ M to 10^−5^ M [83]. They observed substantial alterations up to after culture with 100 µM BPAF and BPA, which is consistent with the results of our experiments. Tyner et al. (2022) found that BPF induces an increase in progesterone production compared to a decrease in BPA, which suggests the upregulation of steroidogenic enzymes after BPF exposure [74]. Their findings are consistent with our findings, which showed elevated levels of progesterone up to 75 µM BPF.

Feng et al. (2016) determined the effect of four bisphenols (BPA, BPAF, BPF, and BPS) on progesterone synthesis after 48 h of cultivation of human adrenocarcinoma cells NCI-H295R [66]. The results from their experiments, as well as our results, indicate a significant effect of bisphenols on steroidogenesis and progesterone production. They noted no significant increase with BPA, but at concentrations of 30, 50, and 70 µM, they noted a significant decrease in progesterone synthesis. We noticed a significant decrease from a concentration of 10 µM and a significant increase in concentrations of 0.05–0.5 µM. In the case of BPAF, they noted a slight increase after cultivation with 0.1 and 1 µM and a significant increase after cultivation with 10, 30, and 50 µM, while the concentration of 70 µM was no longer evaluated, as they marked it as cytotoxic. The effect of bisphenol S was also determined by Amar et al. (2020), using human granulosa ovarian cells as a model system [84]. Progesterone synthesis was significantly stimulated after culture with 10 nM, 100 nM, and 1 µM and significantly inhibited after culture with 10 µM and 50 µM BPS. During our experiments, as mentioned in the text above, we, in contrast to the results of Amar et al. (2020), noted a significant decrease only after cultivation with 25–100 µM and a significant increase after cultivation with 0.1–10 µM. Feng et al. (2018) determined the effect of bisphenol AF on a mouse Leydig cell tumor cell line (mLTC-1) during a 24 h exposure [85]. For their experiment, they chose concentrations 0.1–70 µM BPAF, as concentrations above 80 µM showed cytotoxicity. Progesterone production was not significantly affected by concentrations of 0.1–30 µM; however, from a BPAF concentration higher than 30 µM, progesterone concentration decreased from 56.6 ng/mL (control group) to 38.7 and 30.0 ng/mL (experimental groups 50 µM and 70 µM). During our experiments, after culture with 75 and 100 µM, we noted a significant decrease in progesterone production; however, in concentrations of 0.05–50 µM, in contrast to the study performed on mLTC-1 cells, we noted a significant increase in progesterone production. We demonstrated that BPA and BPS altered steroidogenesis by increasing oestradiol secretion and reducing progesterone secretion. BPS was even more detrimental to progesterone secretion compared to BPA because it induced a reduction at a concentration ten-fold lower than BPA [86]. At a concentration of 10 µM, BPA was able to increase the mRNA expression of StAR, reduce the progesterone level in human ovarian GCs (KGN), and maintain Hsd3b2 (a crucial gene involved in progesterone production) and Cyp11a1 expression levels, which may be the reason for the change in progesterone concentration [87,88]. Our findings show that the BPAF, BPB, BPF, and BPS are not an optimal replacement for BPA in industrial applications since they significantly affect progesterone production, which is an indication of endocrine disruption.

### 4.5. Estradiol

Although bisphenols, especially BPA, are well-known estrogenic endocrine disruptors, inconsistent effects on estradiol (E2) biosynthesis have been determined in scientific publications. Very limited studies are currently available on the effects of BPA on human adrenocarcinoma cells, which are a globally used model system for monitoring changes in steroidogenesis. Data published in the literature from investigations carried out on cellular model systems are as follows: reduction of E2 production at 87.6 µM BPA in luteinized granulosa cells [89], reduction of E2 production at 10 and 100 nM BPA in KGN cells [90] and probably no effect on E2 production at 40–100 µM BPA in KGN cells [91]. In contrast to a reduction or no effect on E2 release, Liu et al. (2021) observed that BPA at a concentration of 10 pM significantly stimulated E2 production in KGN cells [92]. Based on the data from these studies, BPA likely exhibits biphasic effects rather than a classical linear dose-dependent response. In their study, they also identified the underlying mechanism responsible for BPA-induced E2 production in KGN cells. A low concentration (10 pM) of BPA for 24 h significantly up-regulated the CYP19A1 gene, and this effect was mediated by the transcription factor FOXL2. The effect of bisphenol S was also determined by Amar et al. (2020), using human granulosa ovarian cells as a model system [84]. Estradiol synthesis was significantly stimulated after cultivation with 10 nM, 100 nM, 1 µM, and 10 µM and significantly inhibited after cultivation with 50 µM BPS [84], which confirms the biphasic effect of bisphenols determined by our experiments. During their experiments, they also determined the effect of bisphenol S on progesterone synthesis, while, just like during our experiments, they noted a more significant stimulation of estradiol production compared to stimulation of progesterone production. Lin et al. (2021) determined the effect of BPA, BPB, and BPF on the steroidogenesis of H295R human adrenocarcinoma cells [67]. With BPA and BPF, they determined a statistically significant stimulation of estradiol synthesis after cultivation with 6.25 and 25 µM, with BPB at the same concentrations, it was a statistically insignificant stimulation of estradiol synthesis. In our experiments, we noted a significant stimulation of estradiol production with BPA at concentrations of 1–25 µM, with BPB at concentrations of 0.05–0.1 µM, and with BPF at concentrations of 0.01–75 µM.

Bunjakova Mlynarcikova et al. (2021), in contrast to the results from our experiments, noted minimally significant changes in estradiol synthesis in granulosa cells of pigs after exposure to bisphenol A, AF, and F for 24, 48, and 75 h at concentrations of 10^−9^ M to 10^−4^ M [83]. The only statistically significant difference was determined in the reduction of estradiol production after cultivation with the highest concentration (100 µM) of BPAF and BPS, which is in agreement with our results. They noted an insignificant increase after cultivation with 0.001–10 µM BPAF, and 0.001–1 µM BPS. With BPA and BPF, they recorded an insignificant decrease in estradiol synthesis in all bisphenol-exposed experimental groups (10^−9^ M to 10^−4^ M).

Another study in human placental JEG-3 cells examined the effect of BPA on progesterone concentration after 24 and 48 h of exposure. After 24 h of cultivation, they noted a non-significant increase after cultivation with 0.1 µM, a non-significant decrease after cultivation with 1 µM, and a significant decrease after cultivation with 10 and 50 µM. During our experiments on a human adrenal carcinoma cell line, we noted a significant decrease from a concentration of 50 µM and a significant increase in estradiol production at 1–25 µM BPA. After 48 h of cultivation, they noted a non-significant decrease after cultivation with 0.1 µM and a significant decrease after cultivation with 1, 10, and 50 µM. After 48 h, we noted a significant decrease from a concentration of 50 µM and an increase in concentrations of 0.05–25 µM, while at a concentration of 1 µM, there was an insignificant increase in estradiol production. Changes in CYP1A1 and CYP19A1 gene expression in JEG-3 cells caused by BPA exposure, according to [93], may have altered estradiol production and catabolism, leading to aberrant estradiol levels [94]. A significantly reduced expression of CYP19A1 mRNA by 20 µM BPA was also determined in another study [93]. The significant influence of BPA, BPF, BPF, and BPS was also confirmed by another study from 2014, where they determined a significant stimulation of estradiol production [82]. Additionally, it has been reported that BPA may enhance the mRNA transcription of FSHR, StAR, and Cyp19a, which are all involved in the transformation of C19 androgens into C18 estrogens, in a dose-dependent manner. Thus, the overexpression of two genes—FSHR, which produces estradiol, and Cyp19a transcription, which converts T into estrogens—could be the cause of the elevated E2 levels and decreased T synthesis [88]. According to our findings, BPAF, BPB, BPF, and BPS are not suitable substitutes for BPA in industrial applications because they significantly affect the production of estradiol, which is a sign of endocrine disruption.

### 4.6. Testosterone

Inhibition of testosterone production after 24 h of cultivation with BPA was determined by Gonçalves et al. (2018) in TM3 Leydig cells [68]. During their experiment, BPA concentrations of 1, 10, and 100 µM reduced testosterone concentration by 22%, 28%, and 39%, respectively, compared to the control group. These results are in agreement with our results, as we observed a significant decrease in testosterone synthesis by adrenocarcinoma cells in experimental groups 0.1–100 µM after cultivation with BPA. Rosenmai et al. (2014) determined the effect of bisphenol A analogs-bisphenol B, F, and S on testosterone production [82]. The results of their experiments showed that testosterone concentrations decreased with exposure to the test compounds, with BPA showing the strongest effect on androgen levels compared to the other test compounds. During our experiments, they showed the strongest inhibitory effect on testosterone concentration compared to the other tested compounds BPS and BPA. The in vitro and in vivo mechanistic data consistently demonstrated BPB’s capacity to decrease testosterone production and exert an estrogenic-like activity similar to or greater than BPA’s [95].

Studies have also determined the inhibitory effects of bisphenol F and bisphenol S on testosterone secretion by human fetal testes, demonstrating their antiandrogenic effect [96]. Recent in vitro and in vivo studies have determined that bisphenol F inhibits testosterone synthesis, and this inhibition is believed to be due in part to oxidative stress induced by BPF exposure [97,98]. A study on a human breast cancer cell line demonstrated estrogenic and antiandrogenic effects of bisphenol S [99]. Wang et al. (2021) determined a negative correlation between bisphenol A and testosterone concentration in an experiment conducted on 1317 participants aged 6–19 years [100]. Another in vivo study from 2012 conducted on rat testes confirmed the inhibitory effect of bisphenol AF on testosterone concentration, and according to the authors, BPAF-induced inhibition of testosterone production was primarily the result of gene and protein alterations in the testosterone biosynthesis pathway [101]. A study conducted on male rats determined a dose-dependent decrease in testosterone secretion, with a significant (*p* < 0.001) decrease in plasma and intratesticular testosterone observed at a dose of 100 mg/kg BPF [102]. According to Huang et al. (2013), bisphenol A has significant effects on testosterone synthesis in the testes of mice cultured in vitro, while their experiments determined a dose- and time-dependent significant inhibition of testosterone production probably caused by suppression of 3β-HSD, P450c17, and Vimentin [103]. Inhibited androgen synthesis induced by the endocrine disruption caused by bisphenols, specifically disruption of testicular 3β-hydroxysteroid dehydrogenase was also determined by Zhang et al. (2019) [104]. Our findings show that the BPA analogs investigated during our experiments had a considerable impact on testosterone production, indicating endocrine-disruptive activity, and thus are not an appropriate replacement for industrial applications.

### 4.7. Cortisol

The endocrine-disrupting potential of bisphenols and their ability to disrupt cortisol synthesis were also determined in another study. Mustieles et al. (2018) analyzed samples from 172 boys aged 9 to 11 years and determined the correlation between urinary bisphenol A concentration and changes in serum cortisol concentration [105]. Their results suggest the potential of BPA to disrupt the endocrine system during an important period of development. Linear regression models demonstrated that each natural log unit increase in urinary bisphenol A concentration was associated with a 16% decrease in serum cortisol concentration.

Feng et al. (2016) exposed H295R cells to different concentrations of bisphenol A, AF, F, and S for 48 h. They determined the effect of these bisphenols on cortisol synthesis, with concentrations of 1 and 10 µM BPA being comparable to the range of blood levels detected in occupationally exposed individuals [66]. They reported a non-significant decrease in cortisol in the experimental groups exposed to 0.1–10 µM and a significant decrease in cortisol in the 30, 50, and 70 µM experimental groups after 48 h of cultivation with BPA. During our experiments, we also observed a decreasing trend in changes in cortisol production: a non-significant decrease after cultivation with 0.05 µM and a significant decrease after cultivation in the experimental groups exposed to 0.1–100 µM BPA. With BPAF, they determined insignificant stimulation of cortisol production after cultivation with 0.1 µM, insignificant inhibition of cortisol production after cultivation with 1 µM, and significant inhibition of cortisol production after cultivation with 10, 30, and 50 µM. The concentration of 70 µM BPAF was not evaluated due to cytotoxicity. We determined a significant stimulation of cortisol production in experimental groups exposed to 0.05 and 0.1 µM after cultivation with 0.01–100 µM BPAF and a statistically significant decrease after cultivation in experimental groups exposed to 0.5–100 µM. After 48 h of cultivation with BPF, they noted a non-significant decrease in the experimental groups exposed to 0.1 µM, 1 µM, and 30 µM, and a significant decrease in the experimental groups exposed to 10, 50, and 70 µM. During our experiments, in accordance with Feng et al. (2016), who did not observe an increase in the experimental groups exposed to 0.05–100 µM BPF, however, we observed a significant decrease in the experimental groups exposed to 0.05 µM, 0.1 µM, and 10–100 µM BPF. We noted a slight insignificant decrease in the experimental groups exposed to 0.5 and 1 µM. After culturing with bisphenol S, they determined a non-significant inhibition of cortisol production after culturing with 1–10 µM and a significant inhibition of cortisol production after culturing with 10–70 µM. After culturing with BPS, our experiments noted a significant stimulation of cortisol production in the experimental group exposed to 1 µM. In the experimental groups 0.05–0.5 we noted a slightly insignificant stimulation of cortisol production; in the experimental groups 10–100 µM we noted a significant inhibition of cortisol production. Rosenmai et al. (2014) reported that BPA, BPB, and BPS resulted in a significant decrease in cortisol concentration, while BPF resulted in a significant increase in cortisol concentration [82]. Our findings show that the BPA replacements investigated in our study had a considerable impact on cortisol production, a marker of endocrine disruption, indicating that they are not an acceptable replacement.

### 4.8. Future Direction and Challenges

The general population’s exposure to BPA alternatives has increased because they partially replaced BPA in the production of polycarbonates and epoxy resins. Human exposure to BPA alternatives is inevitable as more of them make their way into the market. Thus, public health must evaluate endocrine disruption and analyze the risks associated with new BPA substitutes before their manufacturing [55,106]. The use of animal models to screen novel BPA substitutes is limited by the drawbacks of in vivo testing, which remains the gold standard in toxicology despite their high cost, extended testing duration, and intricate ethical considerations. Hence, in vitro assays offer quicker, less expensive, and more efficient means of screening viable BPA substitutes utilized in industry. The consumer exposure level is emphasized in the current FDA guidelines for premarket submissions for food contact chemicals; nevertheless, specific testing for endocrine disruption is not required [106]. Additionally, it is important to assess the cumulative and possible synergistic effects of different bisphenols [107]. Promising substitutes for some of the in vitro methods are the high-throughput platform applications and computational methods (Tox21, ToxCast,114 PubChem FingerprintSVM,115, and MODELER 9 V7) [106,108].

## 5. Conclusions

Bisphenol A is a high-volume chemical that is extensively used in the production of polycarbonate plastics, epoxy resins, and thermal paper. Because of its endocrine disruptive effects, its use in many consumer items has been restricted or prohibited. Many chemical compounds with comparable chemical structures to bisphenol A have been employed as substitutes in consumer items, such as BPAF, BPB, BPF, and BPS. The manufacturing and application of these analogs are expected to increase in the future. Therefore, we have investigated the impact of BPA, BPAF, BPB, BPF, and BPS on mitochondrial activity and steroidogenesis of human adrenocortical cells. 

Based on the MTT assay, we may conclude that the rank order of mitochondrial toxicities of the five chemicals studied was BPB > BPF > BPAF > BPA > BPS. The most prominent biphasic effect was observed after BPF and BPAF culture since low concentrations of these endocrine disruptors enhanced mitochondrial function, most likely by cell proliferation stimulation. 

Based on the 5-CFDA AM assay, we can conclude that the effect on disrupting the integrity of the cell membrane was in the order of BPB > BPF > BPAF > BPA > BPS. The most pronounced biphasic effect was noted with BPAF, as its low levels stimulated cell membrane integrity, probably stimulation of cell proliferation.

According to our results, we can confirm that human adrenocortical carcinoma cells have a sensitive reaction to bisphenol exposure since we confirmed a significant decrease in the concentration of intracellular cholesterol after exposure to all the bisphenols. The rank order of effect on increasing cholesterol concentration after cultivation with a higher concentration of BPs was BPB > BPA > BPS > BPF > BPAF. A significant decrease in intracellular cholesterol concentration was observed after 24 h of cultivation after exposure to all the bisphenols we monitored. The rank order of effect on a significant decrease in low concentrations of BPs was BPF > BPAF > BPS > BPA > BPB.

BPF followed by BPAF and BPS had the most pronounced effects on stimulating progesterone production. BPB followed by BPA had the least significant effects on the stimulation of progesterone production. BPB, BPS, and BPA had the most pronounced effects on progesterone production inhibition at higher concentrations, BPAF and BPF showed the least significant effects on progesterone production inhibition at higher concentrations. From the point of view of the effect on the secretion of progesterone by adrenocarcinoma cells, we can name bisphenol A as the least biologically effective bisphenol. 

BPF followed by BPA, BPAF, and BPS had the most pronounced effects on the stimulation of estradiol production. BPB had the least significant effects on the stimulation of estradiol production. BPB, BPA, BPS, and BPAF had the most pronounced effects on the inhibition of estradiol production at higher concentrations (in that order), BPF showed the least significant effects on the inhibition of estradiol production at higher concentrations. The least biologically effective BPA analog on estradiol secretion was BPAF. 

We observed a decrease in testosterone concentration after cultivation with BPA, BPAF, BPB, BPF, and BPS in all experimental groups. The most significant decrease was recorded for BPB, BPS, and BPA (in that order). BPF and BPAF showed the least inhibitory effects on testosterone production by adrenocarcinoma cells. The least biologically effective BPA analog on testosterone secretion was BPF.

At high concentrations, the effect of bisphenols on the inhibition of cortisol secretion was manifested in the following order BPA, BPB, BPAF, and BPS. The least pronounced inhibitory effect was observed after cultivation with BPF. With bisphenol AF, we noted the most pronounced biphasic effect (stimulation after cultivation with 0.05 and 0.1 µM and inhibition from a concentration of 1 µM). The least biologically effective BPA analog on cortisol secretion was BPF. 

The current article provides evidence for the endocrine disruptive potential of BPA alternatives, which are nowadays commercially used in many products of everyday use. Based on our findings, we may conclude that BPAF, BPB, BPF, and BPS are not suitable alternatives to BPA since they might possess endocrine disruptive properties; however, more detailed research is needed to clarify the mechanism of action of bisphenols in H295R cells to objectively evaluate the full extent endocrine-disruptive properties.

## Data Availability

Data are contained within the article.
